# A new genus and a new species of microcotylids (Polyopisthocotyla, Platyhelminthes), gill parasite of the pink dentex *Dentex gibbosus* (Teleostei, Sparidae) off Tunisia and notes on Polyopisthocotyla and Monopisthocotyla from *Dentex* spp.

**DOI:** 10.1016/j.ijppaw.2024.101016

**Published:** 2024-11-10

**Authors:** Ilhem Hamdi, Bouchra Benmansour, Mohammed Ahmed, Mehreen Gulsher, Chahinez Bouguerche

**Affiliations:** a Laboratory of Biodiversity, Parasitology and Ecology of Aquatic Ecosystems, Faculty of Sciences of Tunis, University of Tunis El Manar, 2092, Tunis, Tunisia; b Department of Zoology, Swedish Museum of Natural History, Box 50007, SE-104 05, Stockholm, Sweden; c Department of Evolution, Ecology and Behaviour, Institute of Infection, Veterinary and Ecological Sciences, University of Liverpool, Liverpool, L69 7AB, UK; d University of Carthage, Faculty of Sciences of Bizerte, 7021 Zarzouna, Tunisia

**Keywords:** Monogenea, Polyopisthocotyla, *Ktarius* n. gen., Sparidae, DNA barcoding, Mediterranean

## Abstract

The study of the polyopisthocotylan parasites of marine fishes in the western Mediterranean is carried on using an integrative approach combining morphology and DNA barcodes. *Ktarius patrickbrueli* n. gen. n. sp (Polyopisthocotyla, Microcotylidae), from the gills of the pink dentex *Dentex gibbosus* (Teleostei, Sparidae) from the western Mediterranean Sea off Tunisia, is described. Anatomical and morphological features of the new genus are described, and the molecular barcodes for nuclear and mitochondrial markers (28S rRNA and *cox*1) are generated. The new genus is closely related to *Microcotyle* by sharing a symmetrical haptor, inverted question mark-shaped ovary and unarmed vagina. However, *Ktarius* n. gen. can be distinguished from *Microcotyle* and other Microcotylinae taxa by an unarmed male copulatory organ, formed by a long muscular cirrus, a basal layer of concentric muscles, and an elongated thick-walled ejaculatory bulb. A partial 28S rDNA sequence of *K. patrickbrueli* n. gen. n. sp*.* was obtained and found to be distinct from all known microcotylid sequences, with a *p*-distance of 5–13%. A phylogenetic tree constructed from available microcotylid sequences revealed that *K. patrickbrueli* n. gen. n. sp. clustered in a strongly supported clade of Microcotylinae, containing species of *Omanicotyle*, *Bivagina,* and *Microcotyle* confirming its belonging to the Microcotylinae subfamily. The *cox*1 sequences of *K. patrickbrueli* n. gen. n. sp. were highly divergent from the closely related genus *Pauciconfibula* and confirmed its distinction. This new genus is the third polyopisthocotylan genus to be described from sparids of *Dentex*.

## Highlights

• A new genus and species *Katrius patrickbrueli* n. gen n. sp. are described.
• Within Microcotylinae, the new genus is unique by its male copulatory organ.
• The new species shows significant genetic divergence from related taxa.
• Hosts and records of Monopisthocotyla and Polyopisthocotyla from *Dentex* spp. are discussed.

## Graphical abstract

**Figure:**
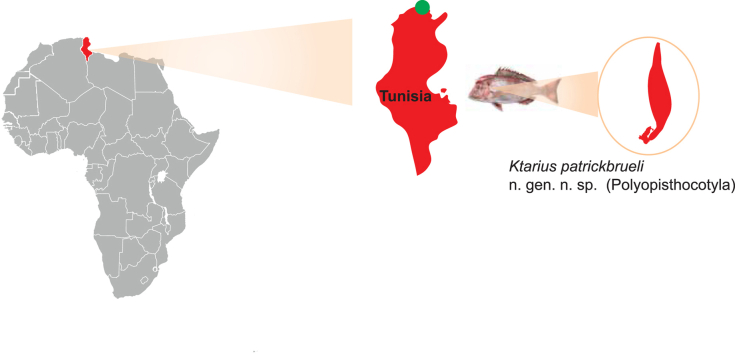

## 1 Introduction

Four species of *Dentex* Cuvier (Sparidae) are known from Mediterranean waters (Neifar, 2008). The large-eye dentex *D. macrophthalmus* (Bloch) and the Morocco dentex *D. maroccanus* Valenciennes are distributed on the southern coasts of the Mediterranean Sea, while the common dentex *D. dentex* Linnaeus and the pink dentex *D. gibbosus* (Rafinesque) are present throughout the Mediterranean Sea (Bauchot and Hureau, 1986). *Dentex gibbosus* is a demersal marine fish from temperate and subtropical habitats (Alves and Vasconcelos, 2012). This sparid, distributed throughout the Mediterranean and east Atlantic coasts (Fischer et al., 1987) is considered a highly praised consumable fish, but it is rarely investigated for its parasites.

Overall, for Monopisthocotyla^1^ Odhner, 1912, six diplectanid species of the genus *Lamellodiscus* Johnston and Tiegs, 1922, and one capsalid of the genus *Encotyllabe* Diesing, 1850 had been reported on *Dentex* spp. worldwide (Euzet et al., 1993; Neifar, 2008; Diamanka et al., 2011a, 2011b). Similarly, reports of Polyopisthocotyla^1^ Odhner, 1912 are scarce and limited to three genera (Table 1). For the latter class, five *Microcotyle* Van Beneden and Hesse, 1863 species were reported on *Dentex* spp. (Euzet et al., 1993; Tokşen and Çilli, 2010; Jun, 2015; Víllora-Montero et al., 2020; Layka et al., 2023), of which three were noted as unidentified species (Euzet et al., 1993; Tokşen and Çilli, 2010; Jun, 2015). One additional microcotylid of the genus *Pauciconfibula* Dillon and Hargis, 1965 and one gotocotylid of the genus *Gotocotyla* Ishii, 1936 are known to occur on *Dentex* spp. (Ktari, 1971; Euzet et al., 1993). Despite most of the previously mentioned taxa occurring in Mediterranean waters (Euzet et al., 1993; Tokşen and Çilli, 2010; Jun, 2015; Víllora-Montero et al., 2020; Layka et al., 2023), to date, only three species have been reported on *Dentex* spp. from Tunisia: *P.* euzeti (Ktari, 1971), *L. euzeti* Diamanka, Boudaya, Toguebaye and Pariselle, 2011 and *L. crampus* Neifar, 2008 (Neifar, 2008; Ktari, 1971; Diamanka et al., 2011).

**Table 1:** Polyopisthocotyla (indicated as P.) and Monopisthocotyla (indicated as M.) known from *Dentex* spp. Parasites for which *Dentex* spp. are type-hosts are indicated in bold.

Host/parasite species	Locality	Type-host/type locality	Source
1 *Dentex canariensis*			
*Lamellodiscus euzeti* Diamanka, Boudaya, Toguebaye and Pariselle, 2011^b^ (M.)	Senegal, ECA.	–	Diamanka et al. (2011a)
2 *Dentex dentex*			
***Microcotyle whittingtoni*** Víllora-Montero, Pérez-del-Olmo, Georgieva, Raga and Montero, 2020 (P.)	Spain, WM.	–	Víllora-Montero et al. (2020)
*Microcotyle* sp 1. a (P.)	Turkey, EM.		Tokşen and Çilli (2010)
*Microcotyle* sp 2. a (P.)	Greece, EM.		Jun (2015)
*Encotyllabe vallei* Monticelli, 1907 (M.)	Mediterranean Sea	*Sparus aurata*/Italy, WM.	Monticelli (1907); Euzet et al. (1993)
*Gotocotyla acanthura* (Parona and Perugia, 1891) (P.)	Mediterranean Sea	*Brama brama*/Italy, WM.	Parona and Perugia (1896); Euzet et al. (1993)
3 *D. gibbosus*			
***Ktarius patrickbrueli*** n. gen. n. sp. (P.)	Tunisia, WM.	–	Present study
***Pauciconfibula euzeti*** (Ktari, 1971) (P.)	Tunisia, WM.	–	Ktari (1971)
*Microcotyle* sp 3. ∗ (P.)	Mediterranean Sea	–	Euzet et al. (1993)
*Encotyllabe vallei* (M.)	Mediterranean Sea	*Sparus aurata*/Italy, WM.	Monticelli (1907); Euzet et al. (1993)
*Gotocotyla acanthura* (P.)	Mediterranean Sea	*Brama brama*/Italy, WM.	Parona and Perugia (1896); Euzet et al. (1993)
***Lamellodiscus euzeti* (M.)**	Tunisia, WM.	*Dentex canariensis*/Senegal, ECA.	Diamanka et al. (2011a)
4 *D. macrophthalmus*			
***Lamellodiscus dentexi*** Aleshkina, 1984 (M.)	Africa, Northwest coast, SEA.	–	Diamanka et al. (2011b)
***Lamellodiscus toguebayei*** Diamanka, Neifar, Pariselle and Euzet, 2011 (M.)	Senegal, ECA.	–	Diamanka et al. (2011b)
***Lamellodiscus vicinus*** Diamanka, Neifar, Pariselle and Euzet, 2011 (M.)	Senegal, ECA.	–	Diamanka et al. (2011b)
***Lamellodiscus triacies*** Diamanka, Neifar, Pariselle and Euzet, 2011 (M.)	Senegal, ECA.	–	Diamanka et al. (2011b)
*Microcotyle erythrini* Van Beneden and Hesse, 1863 (P.)	Syria, EM.	*Pagellus erythrinus*/Brest, NEA.	Van Beneden and Hesse (1863); Layka et al. (2023)
5 *Dentex maroccanus*			
***Lamellodiscus crampus*** Neifar, 2008 (M.)	Tunisia, WM.	–	Neifar (2008)

Abbreviations: ECA, Eastern Central Atlantic. EM, Eastern Mediterranean. NEA, Northeast Atlantic. SEA, South-East Atlantic. WM, Western Mediterranean.

a likely conspecific with *Microcotyle whittingtoni* Víllora-Montero, Pérez-del-Olmo, Georgieva, Raga & Montero, 2020.

b Also reported in Abidjan fish market, Ivory Coast on type host (Diamanka et al., 2011a).

Among metazoan parasites, only two species were reported from *D. gibbosus* as type-host, namely the monopisthocotylan *L. euzeti* (Diplectanidae Monticelli, 1903) found between the secondary gill lamellae of *D. gibbosus* off Dakar, Senegal (Diamanka et al., 2011a); and the polyopisthocotylan *P. euzeti* (Microcotylidae Taschenberg, 1879) from the gills of the previousely mentionned host, collected off the golf of Tunis (Ktari, 1971). However, the co-existence of several congeneric species or from different genera is not uncommon in polyopisthocotylans, and thus the fauna of *D. gibbosus* is yet to be explored. Undoubtedly, integrative taxonomy combining morphology and DNA is the most suitable practice to unveil the complete parasite fauna of these sparids.

In recent years, molecular biology and 'omics' have advanced parasitology research, nonetheless, excessive focus on these methods has led to the neglect of less popular themes. The importance of combining traditional and modern approaches to fully grasp the complexity of the systematics is undisputed (Scholz, 2024). In the last years, an increasing interest in the use of DNA barcoding for species identification and taxonomy was observed in North Africa (Chaabane et al., 2015, 2016a, 2016b, 2017, b; Bouguerche et al., 2019a, c; Derouiche et al., 2019; Bouguerche et al., 2020; Azizi et al., 2021; Bouguerche et al., 2021a; Bouguerche et al., 2022; Lablack et al., 2022; Gastineau et al., 2023; Zedam et al., 2023; Cappelletti and Bouguerche, 2024). For fish parasitic Polyopisthocotyla, in several studies, morphological and molecular features have been used for differentiation at both the intra- and interspecific levels, to define species boundaries, to detect complex cryptic species, and to support their phylogenetic relationship. Interestingly, despite the Polyopisthocotyla being considered hosts specific for a long time (Whittington et al., 2000), both patterns of host specificity (oioxeny and stenoxeny) have been demonstrated, highlighting the valuable insights from DNA barcodes. During a collaborative study of helminth parasites of marine fishes from the western Mediterranean (off Tunisia), aiming at describing and barcoding Polyopisthocotyla, an undescribed Microcotylinae Taschenberg, 1879 species was collected from the gills of *D. gibbosus*. The newly collected specimens differed from the only described polyopisthocotylan from *D. gibbosus* by the presence of an unarmed long muscular cirrus with a basal layer of concentric muscles, and an elongated thick-walled ejaculatory bulb. We used integrative taxonomy combining molecular markers (nuclear and mitochondrial markers; 28S rRNA and *cox*1) and morphology to characterize and provide a formal description of a new genus and its type species.

## 2 Material and methods

### 2.1 Host collection

A total of 57 specimens of *D. gibbosu*s (average size 188.36 ± 50.37) were examined for gill parasites. Fish were collected by local fishermen, from off the coast of Beni Khiar, Tunisia (Latitude: 36° 28′ 9.01″ N, Longitude: 10° 46′ 55.99″ E.) (Division 37.1.1, Western Mediterranean Sea) during the period of July 2018–June 2019. Fish were stored on ice and transferred to the laboratory (Laboratory of Biodiversity, Parasitology and Ecology of Aquatic Ecosystems, Faculty of Sciences of Tunis) shortly after capture. Fish were identified using keys (Fischer et al., 1987; Kullander and Delling, 2012).

### 2.2 Morphological methods

Fish were dissected fresh on the day of purchase and the gills were investigated for parasites under a stereomicroscope. The gills were removed, placed in individual petri dishes, and examined. Newly collected Polyopisthocotyla were heat-killed, fixed in near-boiling saline, and preserved immediately in 80% ethanol for parallel morphological and molecular characterization. Four specimens were processed as hologenophore and paragenophores [sensu Pleijel et al. (2008)]. The hologenophore and paragenophores of Polyopisthocotyla from *D. gibbosus* consists of entire specimens, showing taxonomical features (haptor, male copulatory organ and anatomy at level of ovarian region) and only a small piece of tissues from lateral part of the parasite's body was excised and used for DNA extraction (Fig. 3A).

Polyopisthocotylans were stained with acetic carmine, dehydrated in a graded series of ethanol for 15 min each (70, 96, and 100%), cleared in clove oil, and mounted in Canada balsam (Kennedy, 1979; Bouguerche, 2019). The hologenophore and paragenophores were processed according to the same methods. Polyopisthocotylans were identified on stained whole mounts. Drawings were made through a Nikon Eclipse i80 microscope (Nikon Eclipse N*i*, Nikon, Tokyo, Japan) with DIC (differential interference contrast) (Department of Zoology, Swedish Museum of Natural History, Stockholm, Sweden) and a drawing tube. Drawings were scanned and redrawn on a computer with Adobe Illustrator 2023 (Adobe Inc., San Jose, CA, USA). Measurements are in micrometers and indicated as the range (Ktari, 1971) followed by the number of measurements in parentheses. The type material was deposited at the Swedish Museum of Natural History (SMNH), Stockholm, Sweden.

For clamps nomenclature, we followed Euzet and Marc (1963). For the designation of the ventral and dorsal arms of clamps sclerites, we followed Bouguerche et al., 2021b. We followed the terminology as defined by Combes (2003) to describe the host specificity of a parasite in relation to the relatedness of host species: oioxenic is employed for parasites that exploit a single host species; the parasite is denoted as stenoxenic if it exploits a range of phylogenetically related species and euryxenic if it exploits a range of mutually unrelated species.

### 2.3 Molecular methods

Genomic DNA was extracted from three specimens, and genetic sequence data were generated for two markers: partial sequences of the nuclear 28S rRNA gene (domains D1–D3) and the mitochondrial cytochrome c oxidase subunit 1 gene (*cox*1). Total genomic DNA was isolated using the QIAamp DNA Micro Kit (Qiagen, Hilden, Germany). The specific primers JB3 (forward 5′–TTTTTTGGGCATCCTGAGGTTTAT–3′) and JB4.5 (reverse 5′– TAAAGAAAGAACATAATGAAAATG–3′) were used to amplify a fragment of the *cox*1 gene (Bowles et al., 1995; Littlewood et al., 1997). PCR reaction was performed in 20 μl, containing 1 ng of DNA, 1 × CoralLoad PCR buffer, 3 mM MgCl2, 0.25 mM dNTP, 0.15 μM of each primer, and 0.5 units of Taq DNA polymerase (Qiagen). Thermocycles consisted of an initial denaturation step at 94 °C for 2 min, followed by 37 cycles of denaturation at 94 °C for 30 s, annealing at 48 °C for 40 s, and extension at 72 °C for 50 s. The final extension was conducted at 72 °C for 5 min (Bouguerche et al., 2019a).

A 28S rDNA fragment was amplified using the universal primers C10 (5′–ACCCGCTGAATTTAAGCAT–3′) and D2 (3′–TCCGTGTTTCAAGACGG–5′) (Hassouna et al., 1984). PCR reactions were performed in a final volume of 20 μL, containing: 1 ng of DNA, 16CoralLoad PCR buffer, 3 mM MgCl2, 66 mM of each dNTP, 0.15 mM of each primer, and 0.5 units of Taq DNA polymerase. Thermocycles consisted of an initial denaturation step at 94 °C for 1 min, followed by 40 cycles of denaturation at 94 °C for 30 s, annealing at 60 °C, for 30 s, and extension at 72 °C for 1 min. The final extension was conducted at 72 °C for 7 min (Derouiche et al., 2019). PCR products were visualised on a 1.5% agarose gel, purified and directly sequenced in both directions on a 3730xl DNA Analyzer 96-capillary sequencers (Applied Biosystems) at Eurofins Genomics (https://eurofinsgenomics.eu). Sequences were edited and assembled using CodonCode Aligner software (CodonCode Corporation, Dedham, MA, USA), and compared to the GenBank database content with BLAST. Sequences from three individual polyopisthocotylans were obtained and deposited in GenBank.

### 2.4 Phylogenetic analysis

Phylogenetic analyses were performed using the newly generated sequences of polyopisthocotylans from *D. gibbosus* and those of closely related species available in GenBank (Table 2, Table 3). Alignments for each gene region were constructed separately in AliView (Larsson, 2014). The alignment was trimmed to the shortest sequence. Analysis of phylogeny based on Bayesian inference (BI) was performed using MrBayes 3.2.7 (Huelsenbeck and Ronquist, 2001; Ronquist et al., 2012). Test of best nucleotide substitution model was performed using PAUP (Swofford, 2001) implemented in MrModeltest 2.3 (Nylander, 2004). For both 28S rRNA and *cox*1 genes, BI was run with two random starting trees and four Markov chains, three heated and one “cold” for 2 x 10^6^ generations under the general time reversible model (GTR) with gamma distribution and invariable sites (G + I). Tree sampling was performed at 500 generations intervals. The first 500,000 generations were discarded as burn-ins. The Maximum likelihood (ML) tree was computed from the same dataset. The Nucleotide substitution models for phylogenetic analyses using ML method were selected using MEGA11 (Stecher et al., 2020). The Tamura-Nei model (TN93) and the TN93 with discrete Gamma distribution (TN93+G) (Tamura and Nei, 1993) were selected for the 28S rDNA and the *cox*1 respectively. The ML trees were constructed in MEGA11 with 500 replications. *p*-distances (Kimura, 1980) were computed from the same datasets with MEGA11.

**Table 2:** Collection data for 28S rDNA sequences analysed in this study. Subfamilies are indicated in bold.

Polyopisthocotyla	Host	Locality	GenBank	Source
**Microcotylinae**				
*Ktarius patrickbrueli* n. gen. n. sp.	*Denetx gibbosus*	Tunisia, WM	PQ328200	Present study
*Kahawaia truttae* (Dillon and Hargis, 1965)	*Arripis truttacea*	Australia, EIO	GU263831	Catalano et al. (2010)
*Microcotyle erythrini* Van Beneden and Hesse, 1863	*Pagellus erythrinus*	Spain, WM	MN814848	Víllora-Montero et al. (2020)
*Microcotyle isyebi* Bouguerche, Gey, Justine and Tazerouti, 2019	*Boops boops*	Spain, WM	MN814850	Víllora-Montero et al. (2020)
*Microcotyle sebastis* Goto, 1894	*Sebastes* sp.	North Sea, NEA	AF382051	Olson and Littlewood (2002)
*Microcotyle arripis* Sandars, 1945	*Arripis georgianus*	Australia, EIO	GU263830	Catalano et al. (2010)
*Bivagina pagrosomi* (Murray, 1931)	*Sparus auratus*		Z83002	Olson and Littlewood (2002)
*Omanicotyle heterospina* (Mamaev, 1986)	*Argyrops spinifer*	Oman, WIO	JN602095	Yoon et al. (2013)
*Paracaesicola nanshaensis* Zhou, Li, Liu, Ding and Yuan, 2020	–	–	MH700264	Chou, unpublished
*Diplostamenides* sp.	–	–	MH700263	Chou, unpublished
*Diplostamenides sciaenae* (Goto, 1894)	–	–	FJ432589	Su, unpublished
**Atriasterinae**				
*Sparicotyle chrysophryii* (Van Beneden and Hesse, 1863)	*Sparus aurata*	Algeria, WM	OL679675	Lablack et al. (2022)
*Atrispinum acarne* Maillard and Noisy, 1979	*Diplodus vulgaris*	Algeria, WM	OL679671	Lablack et al. (2022)
**Prostatomicrocotylinae**				
*Pauciconfibuloides amazonica* de Aguiar, Domingues, Silva, Ceccarelli, Adriano and Soares, 2021	*Plagioscion squamosissimus*	Brazil, Tapajos river	MT645075 MT645076	de Aguiar et al. (2022)
*Polylabroides* sp.	–	–	MH700258	Chou, unpublished
*Polylabris* cf. *mamaevi* Ogawa and Egusa, 1980	–	–	MH700591	Chou, unpublished
*Polylabris* sp.	–	–	MH700257	Chou, unpublished
*Polylabris sillaginae* (Woolcock, 1936)	*Sillaginodes punctatus*	Australia, EIO	GU289509	Catalano et al. (2010)
**Anchoromicrocotylinae**				
*Cynoscionicola "branquialis"*	*Umbrina xanti*	Mexico, ECP	AF382050	Olson and Littlewood (2002)
**Metamicrocotylinae**				
*Intracotyle* sp.	–	–	MH700262	Chou, unpublished
**Out group**				
*Choricotyle chrysophryi* Van Beneden and Hesse, 1863	*Pagellus bogaraveo*	Algeria, WM.	OL679693	Lablack et al. (2022)

Abbreviations: EIO, Eastern Indian Ocean. ECP, Eastern Central Pacific. NEA, Northeast Atlantic. WIO, Indian Ocean. WM, Western Mediterranean.

**Table 3:** Collection data for *cox*1 sequences analysed in this study.

Polyopisthocotyla	Host	Locality	GenBank	Source
*Microcotyle caudata* Goto, 1894	“*Sebastes inermis* species complex”	Japan, NWP	LC472528 LC472529	Ono et al. (2020)
*Microcotyle kasago* Ono, Matsumoto, Nitta and Kamio, 2020	*Sebastiscus marmoratus*	Japan, NWP	LC472525 LC472527	Ono et al. (2020)
“*Microcotyle sebastis*” a	*Sebastes schlegelii*	South Korea	DQ412044	Park et al. (2007)
*Microcotyle pacinkar* Kamio and Nitta, 2023	*Sebastes taczanowskii*	Japan, NWP	LC753264 LC753265	Yusuke and Masato (2023)
*Microcotyle algeriensis* Ayadi, Gey, Justine and Tazerouti, 2016	*Scorpaena scrofa*	Spain, NEA	OQ243288 OQ243289	Víllora-Montero et al. (2023)
*Microcotyle merche* Víllora-Montero, Pérez-del-Olmo, Valmaseda-Angulo, Raga and Montero, 2023	*Helicolenus dactylopterus*	Spain, NEA	OQ243286 OQ243287	Víllora-Montero et al. (2023)
*Microcotyle visa* Bouguerche, Gey, Justine and Tazerouti, 2019	*Pagrus caeruleostictus*	Algeria, WM	MK275652 MK275653	Bouguerche et al. (2019a)
*Microcotyle isyebi* Bouguerche, Gey, Justine and Tazerouti, 2019	*Boops boops*	Spain, WM	MN816018 MN816019	Bouguerche et al. (2019b)
*Microcotyle justinei* Ayadi and Tazerouti, 2023	*Apogon imberbis*	Algeria, WM	OR570613	Ayadi and Tazerouti (2023)
*Microcotyle whittingtoni* Víllora-Montero, Pérez-del-Olmo, Georgieva, Raga and Montero, 2020	*Dentex dentex*	Spain, WM	MN816010 MN816011	Víllora-Montero et al. (2020)

“*Microcotyle erythrini*” Van Beneden and Hesse, 1863	*Pagellus erythrinus*	France, WM	AY009159	Jovelin and Justine (2001)
*“Microcotyle erythrini”*	*Pagellus erythrinus*	Spain, WM	MN816012	Víllora-Montero et al. (2020)
*“Microcotyle erythrini”*	*Pagrus pagrus*	Algeria, WM	OL675211	Lablack et al. (2022)
*“Microcotyle erythrini”*	*Pagrus pagrus*	Spain, WM	MN816014	Víllora-Montero et al. (2020)
***Ktarius patrickbrueli* n. gen. n. sp.**	***Dentex gibbosus***	**Tunisia, WM**	**PQ319851** **PQ319852** **PQ319853**	**Present study**
*Pauciconfibula draconis* (Briot, 1904)	*Trachinus draco*	Algeria, WM	MW484928 MW484929	Azizi et al. (2021)

*Pauciconfibula trachini* (Parona and Perugia, 1889)	*Trachinus radiatus*	Algeria, WM	MW484930	Azizi et al. (2021)
	*Trachinus radiatus*	Italy, WM	MW484935	Azizi et al. (2021)
	*Trachinus radiatus*	Tunisia, WM	MW484932	Azizi et al. (2021)

*Pseudaxine trachuri* Parona and Perugia, 1889	*Boops boops*	Algeria, WM	MT666075	Bouguerche et al. (2020)
*Allogastrocotyle bivaginalis* Nasir and Fuentes Zambrano, 1983	*Trachurus picturatus*	Algeria, WM	MN192391	(Bouguerche, 2019c)

Abbreviations: NWP, Northwest Pacific. NEA, Northeast Atlantic. WM, Western Mediterranean.

a from a fish farm. Newly generated sequences are in bold.

## 3 Results

### 3.1 Morphology

**Class Polyopisthocotyla Odhner, 1912**.

**Order Mazocraeidea Bychowsky, 1957**

**Family Microcotylidae Taschenberg, 1879**

**Subfamily Microcotylinae Taschenberg, 1879**

#### 3.1.1 *Ktarius* n. gen.

Zoobank: To comply with the regulations set out in article 8.5 of the amended 2012 version of the International Code of Zoological Nomenclature (ICZN), details of this genus have been submitted to ZooBank with the Life Science Identifier (LSID) zoobank.org:act:FB906F76-23FE-43C6-9076-AA60C86749E9.

With characters of family Microcotylidae, and subfamily Microcotylinae. Body lanceolate. Haptor symmetrical, with numerous clamps arranged in two equal rows. Clamps of *Microcotyle*-type, dissimilar in size, decreasing in size antero-posteriorly; largest clamps examined in middle region with the most posterior clamps smaller. Terminal lappet and terminal anchors absent. Buccal organs muscular, paired, septate, with marginal rows of minute spine-like tubercules. Oesophagus simple without diverticula. Intestinal crura largely co-extensive with vitellaria. Testes numerous, post-ovarian, in intercaecal space. Male copulatory organ unarmed, with an elongated muscular cirrus, with basal layer of concentric muscles. Genital atrium muscular, unarmed. Ejaculatory bulb present. Vagina muscular, dorsal, unarmed. Ovary complex, inverted question mark-shaped. Germinal part of ovary approximately ovoid. Eggs stocky, fusiform, operculated, with one extensive apical filament. Vitelline ducts Y-shaped. Vitellaria not extending into the haptor. Parasites of gills of marine teleosts.

Etymology: the generic name “*Ktarius*” honors the Tunisian Parasitologist Professor Mohamed Hedi Ktari, from University of Tunis El Manar for his leading efforts in studying the systematics and biodiversity of polyopisthocotylans in Northern Africa.

*Type-species*: *Ktarius patrickbrueli* n. gen. n. sp.

#### 3.1.2 *Ktarius patrickbruelin. gen. n. sp.* (Fig. 1, Fig. 2, Fig. 3)

*Zoobank:* To comply with the regulations set out in article 8.5 of the amended 2012 version of the International Code of Zoological Nomenclature (ICZN), details of this species have been submitted to ZooBank with the Life Science Identifier (LSID) zoobank.org:act:A5D04D05-B825-4F11-BACF-820199C1DBED.

*Type*-*host: Dentex gibbosus* (Spariformes: Sparidae), Pink dentex.

*Type locality:* off Beni Khiar, Tunisia, Western Mediterranean.

*Site on the host:* gills lamellae.

*Infection details:* Prevalence: 42.1 % (number of infected fish: 24, number of examined fish: 57). Mean abundance: 2.5 ± 1.9. Mean intensity: 1.04 ± 1.74.

*Deposited material:* Holotype (SMNH-Type-9900), 15 paratypes (SMNH- Type-9901–9915) deposited in the Type collections of the Swedish Museum of Natural History (SMNH), Stockholm, Sweden.

*Paratypes with molecular information:* Body (lacking only a small lateral part) mounted on a slide, excised lateral part used for molecular analysis: hologenophore (SMNH-Type-9901); two paragenophores (SMNH- Type-9902, 9903).

*Representative DNA sequences*: Partial 28S rDNA sequences, 1 sequence, GenBank PQ328200 (SMNH-Type-9901). Partial *cox*1, three sequences, GenBank PQ319851 (SMNH-Type-9901); Q319852 (SMNH-Type-9902); PQ319853 (SMNH-Type-9903).

*Etymology:* named after Patrick Bruel, the French singer-songwriter renowned for his nostalgic song « Au Café des Délices » which evokes Mediterranean charm of Tunisia and a sense of nostalgia. The song’s warmth and connection remind the last author of her time in Sweden, where the cherished tradition of *fika* embodies shared moments of togetherness.

#### 3.1.3 Description

**Fig. 1:**
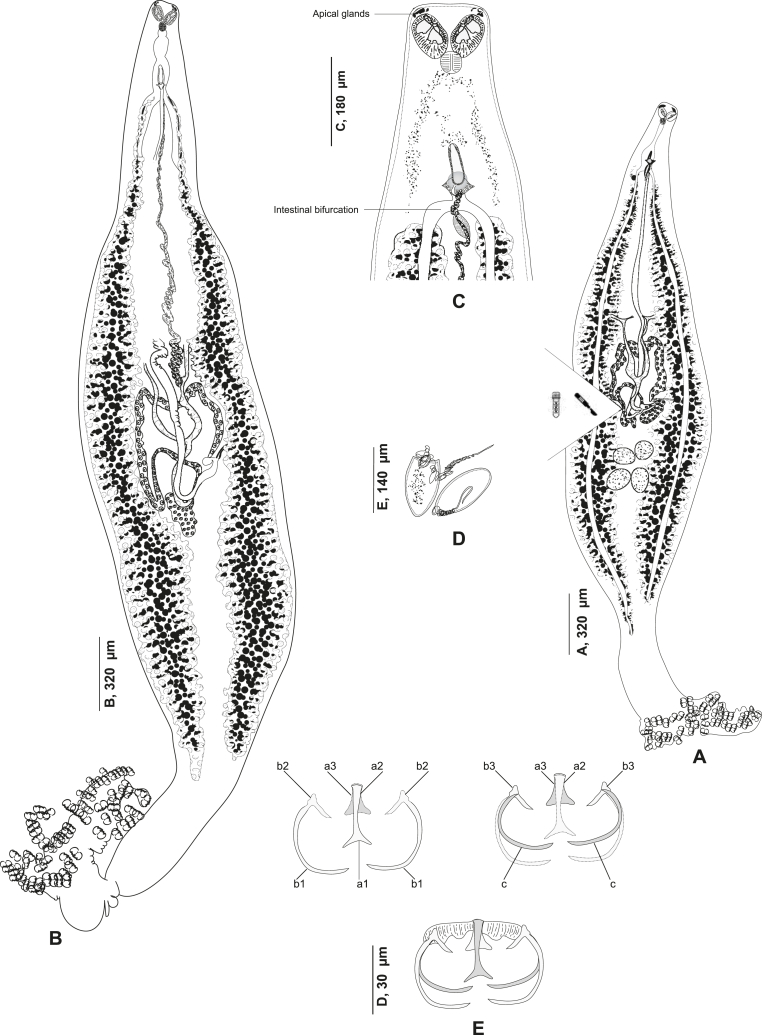
*Ktarius patrickbrueli* n. gen. n. sp. ex *Dentex gibbosus* off Tunisia, Western Mediterranean. A, hologenophores, body lacking only a lateral part; excised used for DNA extraction (Type-9901). B, whole body, (Type-9900). C, anterior part showing apical glands (Type-9915). D, egg (Type-9913). E, disposition of clamp sclerites, dorsal jaw, ventral jaw and a whole clamp (Type-9915).

Based on 17 specimens. Body lanceolate, occasionally rounded anteriorly (Fig. 1A and B), body proper 2983 (2000–4000) long, 573 (320–900) wide. Glandular apical clusters visible in anterior part (Fig. 1 C).

Oral sucker paired, opening laterally, septate, divided by transverse partition into two subequal chambers; 50 (46–58) long, 84 (70–98) wide; rims of oral suckers with numerous minute spine-like tubercules surrounding the anterior and posterior margin (Fig. 1C). Pharynx subspherical, muscular, 42 (40–45) long, 42 (40–45) wide. Oesophagus short, bifurcating at level of the genital atrium. Caeca branched, extending along body; intestinal bifurcation posterior to mal copulatory organ. Caeca not united posteriorly.

**Fig. 2:**
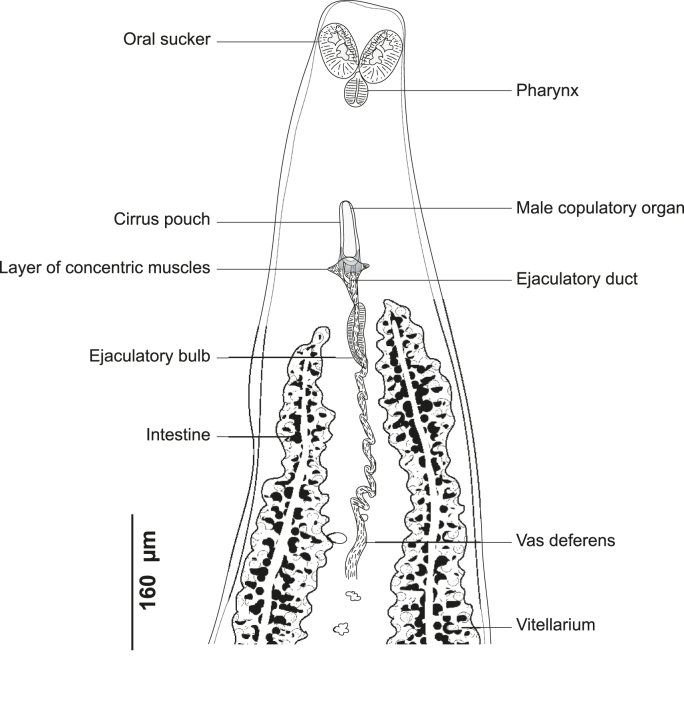
*Ktarius patrickbrueli* n. gen. n. sp. ex *Dentex gibbosus* off Tunisia, Western Mediterranean, anterior end showing relative position of prohaptoral suckers and male copulatory organ (Type-9913).

Testes post-ovarian, in intercaecal space. Vas deferens dorsal, extending along body midline to male copulatory organ (MCO), convoluted in its anterior part before projecting anteriorly in ejaculatory bulb (Fig. 2). Male copulatory organ located at 377 (320–440) from anterior end. Ejaculatory bulb muscular, thick-walled. Cirrus muscular, not sclerotized tube, unarmed, with a basal layer of concentric muscles, cirrus 86 (76–90) long, 25 (18–30) wide. Common genital pore midventral, unarmed.

**Fig. 3:**
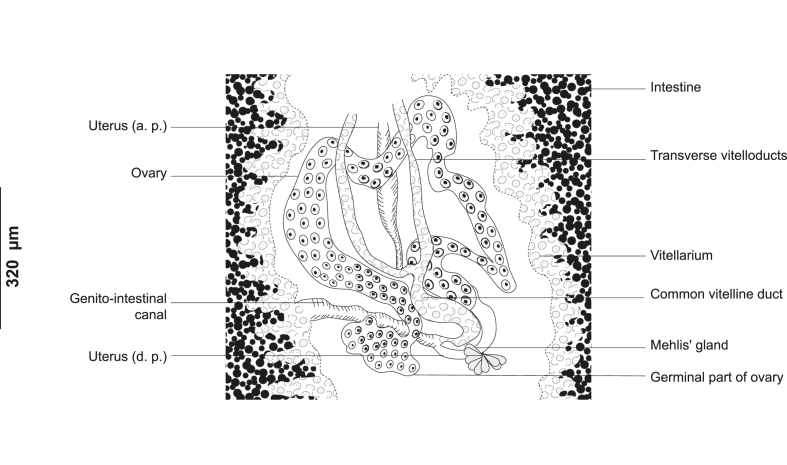
*Ktarius patrickbrueli* n. gen. n. sp. ex *Dentex gibbosus* off Tunisia, Western Mediterranean, detail of the reproductive organs in the region of ovary, ventral view (Type-9913). Abbreviations: a.p., ascendant part; d.p., descendant part.

Ovary complex, pretesticular, intercaecal, irregular, originating in midline, dorsal to vitelline ducts (Fig. 3). Oviduct originating posteriorly from germinal part of ovary. Genito-intestinal canal detaching from right caecum, receiving common vitelloduct. Ootype bounded posteriorly by Mehlis glands. Uterus ascending medio-ventrally and abutting in genital atrium. Vitellarium follicular, co-existing with caeca, extending from genital atrium to posterior part of body. Transverse vitelloducts oblique, short, fusing in midline at level of ovary forming common vitelline duct. Common vitelline duct projecting into oviduct. Vagina mediodorsal, unarmed, 32 (30–35) wide, opening more posteriorly than genital atrium, at 795 (620–960) from anterior end. Eggs fusiform (Fig. 1D), with one long polar filament; 140 long, 65 wide (n = 2).

Haptor triangular, tapering regularly, bearing numerous clamps; 946 (720–1210) long. Clamps arranged in two sub-equal rows. Clamps dissimilar in size, decreasing in size antero-posteriorly, largest clamps examined in middle region; largest clamps 61 (50–72) long, 40 (38–45) wide. Clamps of *Microcotyle*-type.

Clamps consisting of two opposable jaws, anterior jaw and posterior jaw (Fig. 1E). Ventral arm of median spring *a* T-shaped, long; distal part of *a* with short branches of equal size. Dorsal arm of median spring *a* visibly shorter than its ventral arm, T-shaped, distally broad. Ventral arm of ventral jaw consisting of two lateral sclerites *b2*, dorsal arm *b3* shorter and curved inwards; *b3* not reaching dorsal arm of median spring. Dorsal jaw sclerites *c* reaching midline on distal side. Muscles connecting *a* and *b2* present on proximal side.

#### 3.1.4 Differential diagnosis

*Ktarius* n. gen. can be differentiated from the other 24 genera in the subfamily Microcotylinae based on the vaginal number and by the armature of the male copulatory organ. Based on vaginal number, genera of the subfamily Microcotylinae can be attributed to two groups: the bivaginate group (with paired vaginae) and the univaginate group (with a single vaginae). By having a single medio-dorsal vagina, *Ktarius* n. gen. is attributed to the second group.

The species belonging to the following genera all possess a single, unarmed vagina: *Atriostella* Unnithan, 1971; *Caballeraxine* Lebedev, 1972; *Diplostamenides* Unnithan, 1971; *Gamacallum* Unnithan, 1971; *Jaliscia* Mamaev et Egorova, 1977; *Magniexcipula* Bravo-Hollis, 1981; *Paramicrocotyloides* Rohde, 1978; *Paranaella* Kohn, Baptista Farias et Cohen, 2000; *Pauciconfibula* Dillon et Hargis, 1965; *Polymicrocotyle* Lamothe-Argumedo, 1967; *Pseudoaspinatrium* Mamaev, 1986; *Sciaenacotyle* Mamaev, 1986; and, *Solostamenides* Unnithan, 1971 (Yoon et al., 2013). However, *Ktarius* n. gen. can be distinguished from the previously mentioned genera by its muscular unarmed cirrus with a basal layer of centric muscles and an elongated ejaculatory bulb.

Species belonging to *Monomacracanthus* Mamaev, 1986 and *Sebasticotyle* Mamaev et Egorova, 1977 possess a single armed vagina (Yoon et al., 2013). *Ktarius* n. gen. is distinguished from these genera by having an unarmed vagina. *Microcotyle* also have a single unarmed vagina. However, *Ktarius* n. gen. can be distinguished by lacking the characteristic inverted heart-shaped atrium proper, with numerous conical spines, which is well known in *Microcotyle* spp.

All remaining genera in the following section possess paired vaginae (Yoon et al., 2013). *Ktarius* n. gen. can be distinguished from *Diplasiocotyle* Sandars, 1944; *Lutianicola* Lebedev, 1970; *Neobivagina* Dillon et Hargis, 1965; *Pseudobivagina* Mamaev, 1986; and *Pseudoneobivagina* Mamaev, 1986 by its a single unarmed medio dorsal vagina (*vs*. paired unarmed vaginae). Similarly, *Ktarius* n. gen. can be differentiated from *Bivagina* Yamaguti, 1963; *Omanicotyle* Yoon, Al-Jufaili, Freeman, Bron, Paladini and Shinn, 2013; *Kahawaia* Lebedev, 1969; and *Neobivaginopsis* Villalba, 1987 by the possession of a single unarmed medio dorsal vagina (*vs*. paired armed vaginae).

### 3.2 Molecular characterisation

#### 3.2.1 28S analysis

One new sequence (923 pb) was generated for *K. patrickbrueli* n. gen. n. sp. The BLAST search performed using the newly generated sequence did not match any other sequences available in GenBank. A tree was built based on the alignment consisting of 24 sequences of 23 species representing 15 genera of Microcotylidae, with *Choricotyle chrysophryi* Van Beneden and Hesse, 1863 (Diclidophoridae Cerfontaine, 1895) as the outgroup (Table 2). There was a total of 745 positions in the final dataset. The alignment of the 28S rDNA dataset for the family Microcotylidae included representatives of 5 subfamilies: Microcotylinae Taschenberg, 1879, Atriasterinae Maillard and Noisy, 1979, Metamicrocotylinae Yamaguti, 1968, Anchoromicrocotylinae Bravo-Hollis, 1981, Prostatomicrocotylinae Yamaguti, 1968. The BI and the ML method led to similar topologies and only the BI tree is shown.

**Fig. 4:**
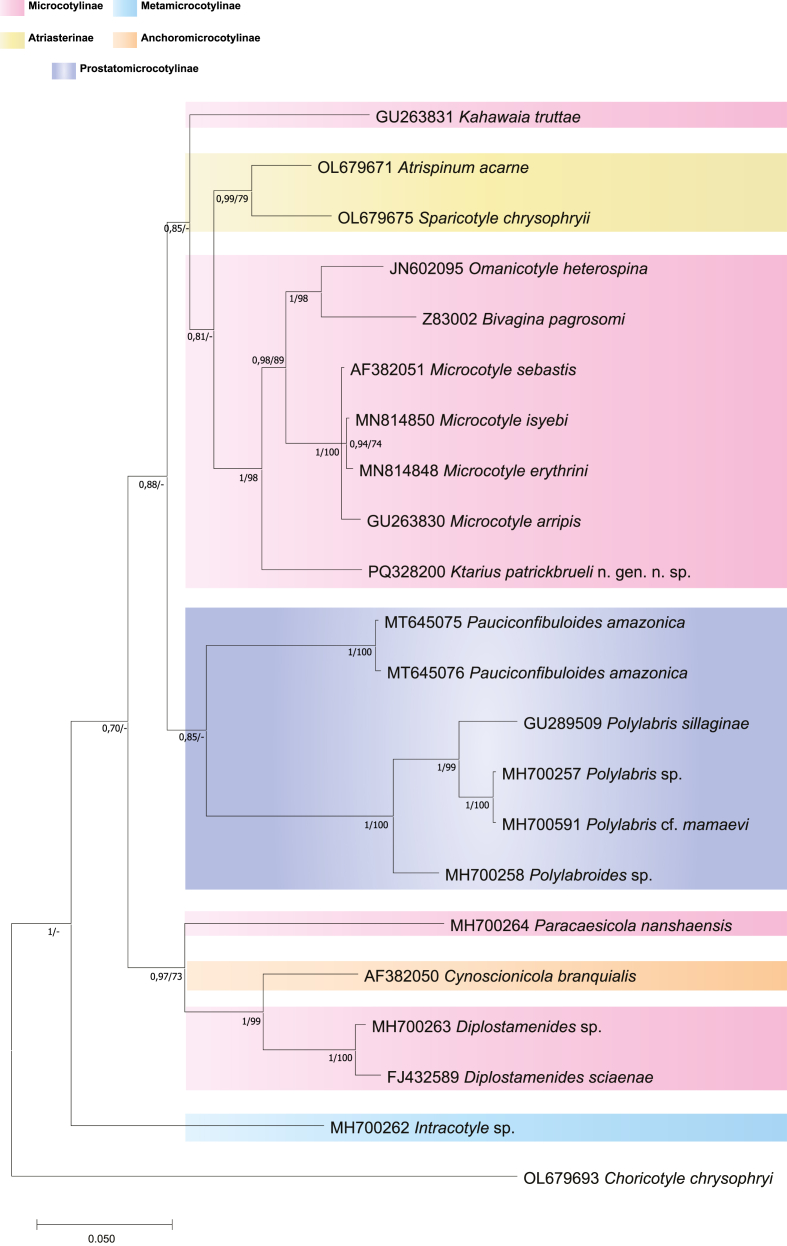
Bayesian topology based on partial 28S ribosomal DNA sequences of Microcotylidae. There was a total of 745 positions in the final dataset. Outgroup: *Choricotyle chrysophryi* (Diclidophoridae). GenBank accession numbers precede species names. The support values are included below the nodes as follows: posterior probabilities for BI analyses, followed by bootstraps for the ML analyses (posterior probabilities >0.90 and bootstrap scores >70 are considered well-supported; posterior probabilities and bootstrap scores <0.70 and 70, respectively, are not shown and are represented by a dash). The scale-bar indicates the expected number of substitutions per site.

The tree resulting from the BI analysis is shown in Fig. 4 along with the statistical support from the ML analysis. *Ktarius* n. gen. along with the genera *Microcotyle*, *Bivagina,* and *Omanicotyle* clustered within the Microcotylinae clade. The Atriasterinae represented by 3 genera (*Sparicotyle* Mamaev, 1986; *Atrispinum* Euzet and Maillard, 1974; *Atriaster* Lebedev and Parukhin, 1969); and the Prostatomicrocotylinae represented by 2 genera (*Pauciconfibuloides* de Aguiar, Domingues, Silva, Ceccarelli, Adriano and Soares, 2021 and *Polylabris* Euzet and Cauwet, 1967 ) were sister clades. Out of the 5 subfamilies, only two were monophyletic: Atriasterinae and Prostatomicrocotylinae, represented both by 3 genera. The Microcotylinae represented by 7 genera was herein weakly resolved and exhibiting a polyphyly. However, *Ktarius* n. gen. clustered with *Bivagina*, *Omanicotyle* and the type-genus *Microcotyle* clearly supporting that our new species is a member of the subfamily Microcotylinae.

For the partial 28S, the evolutionary divergence estimates were compared between the new genus and the 20 microcotylid sequences retrieved from GenBank and found to vary from 5% to 14% (see Supplementary Table S1). The genetic divergences among *Ktarius* n. gen. and other Microcotylinae varied between 5 and 6% (divergence to *Microcotyle*) and 14% (divergence to *Paracaesicola* Zhou, Li, Liu, Ding and Yuan, 2020). The genetic divergence to members of Atriasterinae (*Sparicotyle* and *Atrispinum*) and of Anchoromicrocotylinae (*Cynoscionicola* Price, 1962) was 7% and 11% respectively. The genetic divergence to members of Prostatomicrocotylinae was as follows: divergence to *Pauciconfibuloides* 10–11%; divergence to *Intracotyle* Mamaev, 1986, 11%; divergence to *Polylabroides* Mamaev and Parukhin, 1976 and to *Polylabris* Euzet and Cauwet, 1967, 11% and 13% respectively.

#### 3.2.2 *Cox*1 analysis

**Fig. 5:**
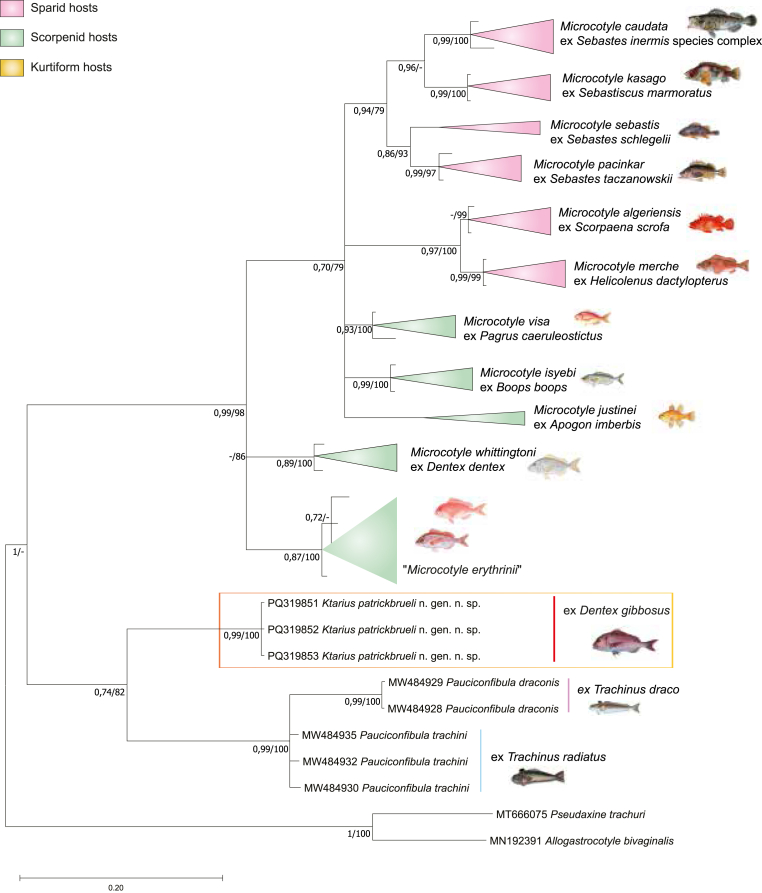
Bayesian topology based on partial *cox*1 for Microcotylinae. Outgroup: *Allogastrocotyle bivaginalis* and *Pseudaxine trachuri* (Gastrocotylidae). GenBank accession numbers precede species names. The support values are included below the nodes as follows: posterior probabilities for BI analyses, followed by bootstraps for the ML analyses (posterior probabilities >0.90 and bootstrap scores >70 are considered well-supported; posterior probabilities and bootstrap scores <0.70 and 70, respectively, are not shown and are represented by a dash). The scale-bar indicates the expected number of substitutions per site.

Partial *cox*1 (412–429 nt) sequences were generated for three isolates. The newly generated *cox*1 of sequences microcotylids ex *D. gibbosus* were analysed together with 22 published sequences for *Microcotyle* spp. and 6 sequences for *Pauciconfibula* spp. The gastrocotylids *Allogastrocotyle bivaginali*s Nasir and Fuentes Zambrano, 1984 and *Pseudaxine trachuri* Parona and Perugia, 1896 were used as outgroups (Table 3). The tree resulting from the BI analysis is shown in Fig. 5 together with the statistical support from the ML analysis.

Microcotylidae clustered in the same clade, distinct from the outgroup. Isolates of *Microcotyle* spp., mainly from sparid and scorpaenid hosts clustered in highly supported clades. Similarly, the *M. “erythrini”* clade with the isolates ex *Pagellus erythrinus* (Linnaeus) and isolates ex *Pagrus pagrus* (Linnaeus) was highly supported. Since this clade does not include sequences of *M. erythrini* Van Beneden and Hesse, 1863 *sensu stricto* from the type-host and type locality (Northeast Atlantic), we refer to it herein as “*M. erythrini*”.

The newly generated sequences of the microcotylids from *D. gibbosus* clustered in a distinct clade, with *Pauciconfibula* as a sister clade. All sequences of *P. trachini* (Parona and Perugia) and *P. draconis* (Briot, 1904) nested within a monophyletic clade. *Pauciconfibula trachini* from the type-host *Trachinus radiatus* Cuvier off three Mediterranean localities (Algeria, Tunisia, and Italy) clustered in a robust clade whereas *P. draconis* from the type-host *T. draco* Linnaeus clustered in a distinct and well-supported clade, supporting thus *P. trachini*, *P. draconis* and *K. patrickbrueli* n. gen. n. sp. as distinct species.

The BLAST search performed using each of the three newly generated *cox*1 sequences did not match any other monogenean sequences available in GenBank. The newly generated *cox*1 sequences were identical between them, and presented evolutionary divergence estimates that were compared between the new species and with 28 microcotylid sequences retrieved from GenBank and found to vary from 21 to 27% (see Supplementary Table S2).

Within *Microcotyle*, intraspecific variations were low and varied between 1 and 2% for *M. pacinkar* Kamio and Nitta, 2023 and *M. visa* Bouguerche, Gey, Justine and Tazerouti, 2019, *M. whittingtoni* Víllora-Montero, Pérez-del-Olmo, Georgieva, Raga and Montero, 2020 and *M. erythrini* respectively. The highest intraspecific variation was among isolates of *M. caudata* Goto, 1894 (3%). Within *Pauciconfibula*, intraspecific variations were noted only for *P. trachini* (2%) whereas isolates of *P. draconis* showed no variations. Intrageneric variations withing *Microcotyle* varied between 4 and 19% and did not exceed 9% withing *Pauciconfibula*. The intergeneric variations between the previously mentioned genera varied between 21 and 28%. Similarly, the intergeneric divergence between *Ktarius* n. gen. and *Microcotyle* and *Pauciconfibula* ranged from 20 to 25 % and from 19 to 21 % respectively.

## 4 Discussion

### 4.1 *Ktarius patrickbrueli* n. gen. n sp.

*Ktarius patrickbrueli* n. gen. n. sp. can be differentiated from the other species of the 24 genera in the subfamily Microcotylinae based on the male copulatory organ and the number and armature of vagina. In *K. patrickbrueli* n. gen. n. sp., in addition to a single unarmed mediodorsal vagina, there is an unarmed muscular cirrus, with a basal layer of centric muscles and an elongated thick-walled ejaculatory bulb. This feature cannot be accommodated into any of the established genera in the Microcotylidae. Due to the lack of a prostatic system in the copulative organ, we place the parasite collected herein on *D. gibbous* among the Microcotylinae. Within this subfamily, the polyopisthocotylan reported herein has a unique male copulatory organ as described above, which allows it to be considered a new species, a type of a new genus. With the erection of *Ktarius* n. gen., the number of genera belonging to the Microcotylinae increases to 25.

One of the useful characteristics in the classification of Polyopisthocotyla is undoubtedly the hard structures i.e. the spines of genital openings. Hence, genera with unarmed male and female genital openings can be taxonomically challenging (Azizi et al., 2021). Among Microcotylinae possessing unarmed male and female genital openings, some *Pauciconfibula* species resemble morphologically *Ktarius* n. gen. To date, *Pauciconfibula* includes six valid species: *P. trachini*, *P. draconis*, *P. pogoniae* (MacCallum, 1913), *P. euzeti*, *P. gallieni* (Euzet and Ktari, 1971) and *P. subsolana* (Chisholm et al., 1991; Briot, 1904; Parona and Perugia, 1889; MacCallum, 1913; Euzet and Ktari, 1971; Ktari, 1971; Chisholm et al., 1991).

The *cox*1 phylogenetic analysis demonstrates that *K. patrickbrueli* n. gen. n. sp. and *Pauciconfibula* spp. form distinct clades, with an intergeneric divergence between *Ktarius* n. gen. and *Pauciconfibula* spp. (19–21%) that significantly exceeds the intrageneric divergence previously reported within *Pauciconfibula* spp. (9–10%) (Azizi et al., 2021) and those observed within other Microcotylidae genera, such as *Microcotyle* (4–19%). These results indicate that *K. patrickbrueli* n. gen. n. sp. cannot be accommodated within the genus *Pauciconfibula* and thus merits its assignment to a new genus, *Ktarius* n. gen. The phylogenetic distinction is further reinforced by morphological differences. *Ktarius patrickbrueli* n. gen. n. sp. is characterized by an elongated cirrus with a basal layer of concentric muscles and an elongated ejaculatory bulb, features absent in *Pauciconfibula* spp. and in *P. euzeti*. Furthermore, *Pauciconfibula* lacks both an elongated cirrus and an ejaculatory duct (Briot, 1904; Parona and Perugia, 1889; MacCallum, 1913; Euzet and Ktari, 1971; Ktari, 1971; Chisholm et al., 1991; Azizi et al., 2021). These combined phylogenetic and morphological findings underscore the distinct status of *K. patrickbrueli* n. gen. n. sp. within the Microcotylidae family.

The erection of this new genus was also supported by the 28S rDNA phylogeny, which placed *Ktarius* n. gen. in the microcotylid clade, but with a high genetic difference from all other ingroup microcotylids (5–8%). Overall, the resulting topology for 28S rDNA phylogeny was in agreement with the classification of species into genera, with all genera monophyletic with high support (Fig. 4).

The 28S rDNA phylogeny was congruent with previous phylogenetic analyses that identified *Omanicotyle*, *Bivagina* and a clade of *Microcotyle* species as members of the Microcotylinae subfamily (Olson and Littlewood, 2002; Yoon et al., 2013; Zhou et al., 2020; Lablack et al., 2022). However, other members of the subfamily Microcotylinae did not form a monophyletic group, such as *Diplostamenides*, *Paracaesicola* that clustered with *Cynoscionicola* member of Anchoromicrocotylinae. The polyphyly of Microcotylinae was previously highlighted (Zhou et al., 2020; Lablack et al., 2022) and the possibility of the Microcotylinae likely requiring division into smaller subfamilies is not to be ruled out (Zhou et al., 2020). However, such a conclusion will require a more robust phylogenetic analysis, based on sequence data for additional taxa and examination of representatives of the previously mentioned taxa.

The unarmed genital atrium, vaginal pore and MCO are shared characters with the closely related genus *Pauciconfibuloides* (de Aguiar et al., 2022) to which we add the fusiform body with a triangular haptor with numerous clamps. However, the two genera can be easily distinguished by the prostatic system being lacking in *Pauciconfibula* (de Aguiar et al., 2022). This systematic scheme concerning Microcotylinae and Prostamicrocotylinae is well supported by the 28S rDNA molecular analysis given here, with *K. patrickbrueli* n. gen. n. sp. and *Pauciconfibuloides amazonica* clustering in different clades (Fig. 4). *Pauciconfibuloides* clustered among Prostatomicrocotylinae along with *Polylabroides* and *Polylabris*, and this well supported by morphologic characters of these genera as they share the possession of a prostatic system (Yamaguti, 1968; Mamaev, 1986; de Aguiar et al., 2022).

Likewise, *K. patrickbrueli* n. gen. n. sp. clustered within Microcotylinae, in which all members lack a prostatic system (Mamaev, 1986), along with *Microcotyle, Bivagina* and *Omanicotyle*. The separation of these microcotylids into different genera in the phylogenetic analysis is also strongly backed by morphologic features, as only the genera *Bivagina* and *Omanicotyle* have an unarmed genital atrium and no differentiated cirrus but separated on the size and armature of their vaginae (Yoon et al., 2013), and *Microcotyle* is well known for its genital atrium that comprises an anterior atrium proper and two posterior ‘‘pockets’’, with the atrium proper roughly shaped as inverted heart, bearing numerous conical spines (Bouguerche et al., 2019a, b). This supports the separation between *Ktarius* n. gen. and the previously mentioned microcotylid genera.

### 4.2 Monopisthocotyla and Polyopisthocotyla from *Dentex* spp.

*Dentex* spp. are hosts for different Monopisthocotyla and Polyopisthocotyla (Table 1). *Dentex* spp. had been reported as type-hosts for members of both classes. For instance, *D. dentex* and *D. gibbosus* are type-hosts for only polyopisthocotylan microcotylids (Ktari, 1971; Víllora-Montero et al., 2020) whilst *D. canariensis*, *D. macrophthalmus* and *D. maroccanus* are type-hosts for monopisthocotylan diplectanids (Neifar, 2008; Diamanka et al., 2011a, b). These sparids are highly likely hosts for other taxa yet to be described, as the occurrence of polyopisthocotylans and monopisthocotylans in the same sparid hosts in common (Kouider El Ouahed-Amine, 1998).

There is a record of a polyopisthocotylan on *D. macrophthalmus*, *M. erythrini* from Lattakia, Syria, eastern Mediterranean (Layka et al., 2023). As the hosts differ, *M. erythrini sensu* Layka et al., 2023 is likely an undescribed *Microcotyle* species. This hypothesis is well supported as previous studies combining morphology and DNA demonstrated that *M. erythrini* is a species complex (Bouguerche et al., 2019a, b; Víllora-Montero et al., 2020). Similarly, *Microcotyle* sp. reported on *D. gibbous* (Euzet et al., 1993) is yet to be described and characterized.

Other remarkable records of gill parasites on *Dentex* spp. is *Gotocotyla acanthura* (Parona and Perugia, 1896) reported on *D. dentex* and *D. gibbosus* (Euzet et al., 1993). The type-host of *G. acanthura* is a bramid fish, the Atlantic pomfret *Brama brama* (Bonnaterre) (Parona and Perugia, 1896), and the conspecificty of the two populations is yet to be demonstrated using integrative taxonomy. In contrast, the records of the capsalid monopisthocotylan *Encotyllabe vallei* Monticelli, 1907 reported on *D. dentex* and *D. gibbosus* are likely accurate as previous studies demonstrated with DNA sequences the stenoxenic specificity of *E. vallei* and demonstrated its occurrence on different sparid hosts (Lablack et al., 2022; Zedam et al., 2023). However, considering the available data, there is no clear pattern for host specificity for Polyopisthocotyla nor for Monopisthocotyla, and DNA sequences generated in the context of integrative taxonomy, preferably with morphometrical and anatomical data and hologenophores are warranted.

## 5 Conclusion

This study provides insights into the diversity of polyopisthocotylan parasites in the western Mediterranean, highlighting the importance of an integrative approach. Based on morphological and molecular evidence, a new genus of microcotylid polyopisthocotylans is erected to accommodate a new species, *Ktarius patrickbrueli* n. gen. n. sp. from the poorly studied sparid fish, *Dentex gibbosus*. The distinct anatomical and molecular characteristics of this new genus not only differentiate it from closely related taxa, such as *Pauciconfibula* and *Microcotyle*, but also underscore the ongoing need for further taxonomic and phylogenetic studies within this group. With the identification of *K. patrickbrueli* n. gen. n. sp. as the third polyopisthocotylan genus recorded from sparids of *Dentex*, this work contributes to the broader knowledge of parasites fauna of *Dentex* spp. and emphasizes the potential for discovering new species in underexplored regions of Northern Africa.

## CRediT authorship contribution statement

**Ilhem Hamdi:** Writing – review & editing, Writing – original draft, Visualization, Validation, Methodology, Investigation, Formal analysis, Data curation. **Bouchra Benmansour:** Writing – review & editing, Validation, Supervision, Resources, Project administration, Methodology, Investigation, Funding acquisition, Conceptualization. **Mohammed Ahmed:** Methodology, Data curation. **Mehreen Gulsher:** Data curation. **Chahinez Bouguerche:** Writing – review & editing, Writing – original draft, Supervision, Resources, Project administration, Methodology, Investigation, Funding acquisition, Formal analysis, Data curation, Conceptualization.

## Declaration of competing interest

On behalf of the co-authors, I am stating that there is no conflict of interest.

## Footnotes

1 For high-level terminology of parasites, we followed the systematics of Brabec et al. (2023) who elevated the former subclasses of "Monogenea" to the level of classes, and we use the classes Monopisthocotyla and Polyopisthocotyla.
